# Modeling and Analysis of Environmental Electromagnetic Interference in Multiple-Channel Neural Recording Systems for High Common-Mode Interference Rejection Performance

**DOI:** 10.3390/bios14070343

**Published:** 2024-07-15

**Authors:** Gang Wang, Changhua You, Chengcong Feng, Wenliang Yao, Zhengtuo Zhao, Ning Xue, Lei Yao

**Affiliations:** 1 School of Microelectronics, Shanghai University, Shanghai 200444, China; wanggang@shu.edu.cn; 2 Zhangjiang Laboratory, Shanghai 200031, China; 3 State Key Laboratory of Transducer Technology, Aerospace Information Research Institute (AIR), Chinese Academy of Sciences, Beijing 100190, China; youchanghua18@mails.ucas.ac.cn; 4 Institute of Neuroscience, State Key Laboratory of Neuroscience, CAS Center for Excellence in Brain Science and Intelligence Technology, Chinese Academy of Sciences, Shanghai 200031, China; ccfeng2020@ion.ac.cn (C.F.); zhaozt@ion.ac.cn (Z.Z.); 5 Shanghai Mtrix Technology Co., Ltd., Shanghai 200031, China; yaowenliang@mtrix.cn; 6 Lingang Laboratory, Shanghai 200031, China; xueningxn@lglab.ac.cn

**Keywords:** electromagnetic interference (EMI), neural recording system, multiple-channel, equivalent circuit model

## Abstract

Environmental electromagnetic interference (EMI) has always been a major interference source for multiple-channel neural recording systems, and little theoretical work has been attempted to address it. In this paper, equivalent circuit models are proposed to model both electromagnetic interference sources and neural signals in such systems, and analysis has been performed to generate the design guidelines for neural probes and the subsequent recording circuit towards higher common-mode interference (CMI) rejection performance while maintaining the recorded neural action potential (AP) signal quality. In vivo animal experiments with a configurable 32-channel neural recording system are carried out to validate the proposed models and design guidelines. The results show the power spectral density (PSD) of environmental 50 Hz EMI interference is reduced by three orders from 4.43 × 10^−3^ V^2^/Hz to 4.04 × 10^−6^ V^2^/Hz without affecting the recorded AP signal quality in an unshielded experiment environment.

## 1. Introduction

Understanding how the human brain works has been a scientific quest and research hot spot in recent decades [1,2,3]. Electrophysiological multiple-channel neural recording system is a popular and powerful research tool in brain research due to its high spatial-temporal resolution compared to computed tomography (CT), functional magnetic resonance imaging (fMRI), magnetoencephalography (MEG), and so on [4]. One problem in such neural recording systems is that the recording system is quite vulnerable to environmental electromagnetic interferences (EMI) [5] since the bandwidth of the neural signals we are interested in is normally between 0.1 Hz to 10 kHz [6] and the impedance of the recording node is quite high up to several mega Ohms [7]. A lot of environmental EMI falls in this bandwidth, such as 50/60 Hz power line interference (PLI), harmonics of nearby electrical equipment and even the electrostatic discharge pulses (ESD) events brought by the experimental animals [8,9,10,11]. Moreover, high node impedance would result in a large portion of EMI voltage on the node, which aggravates this problem. To address this problem, a conventional and effective approach is to add a metal shield around the experimental setup using a Faraday cage or aluminum foils [12,13]. However, this approach is not always feasible, particularly for freely moving animal experiments and clinic applications in the future. There are a few previous works trying to reduce the influence of the EMI by improving the CMRR of the recording IC in the multiple-channel neural recording system [10,14,15], but the influence of the electrode configuration and mismatch is not included in these works, leading to an incomplete optimization model for system common-mode rejection ratio (SCMRR) in a multiple-channel neural recording system.

**Figure 1:**
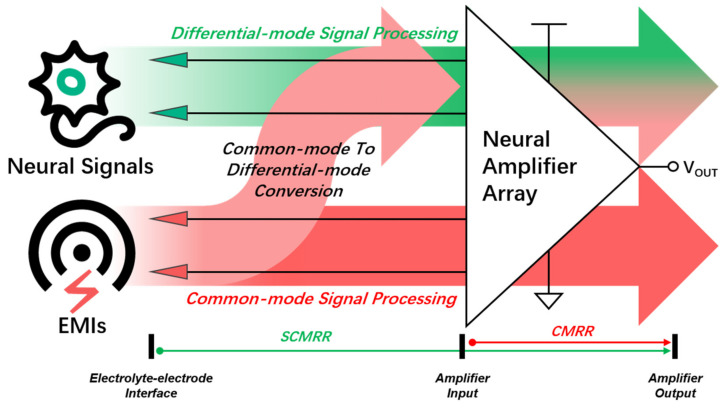
Transmitting relationship of neural signals and EMIs from the electrolyte–electrode interface to neural amplifier array input.

Figure 1 shows a typical system diagram of a multiple-channel neural recording system including electrodes, neural amplifier array, neural signals, and environmental EMIs. To amplify the weak extracellular neural signal (~μV level) between the recording electrode and reference electrode, a differential amplifier is used to provide differential gain and suppress common-mode EMI. Ideally, the common-mode EMI will be suppressed by the differential amplifier. However, due to different configurations of signal/reference/ground electrodes and impedance mismatch of the electrodes, part of the common-mode EMI will be converted to differential-mode EMI before going to the differential amplifier. Thus, a complete modeling and thorough analysis of the EMI for the neural recording system, including the recording electrodes and the recording IC, is required to improve the SCMRR. This paper is organized as follows: Section 2 gives complete definitions including neural signals and EMIs from electrolyte–electrode interface. Section 3, Section 4 and Section 5 gives detailed circuit modes for neural signal path, common-mode EMI path and differential-mode EMI path, respectively. Section 6 shows the in vivo experiment results and Section 7 gives the conclusion.

## 2. Neural Signals and EMIs from Electrolyte–Electrode Interface

**Figure 2:**
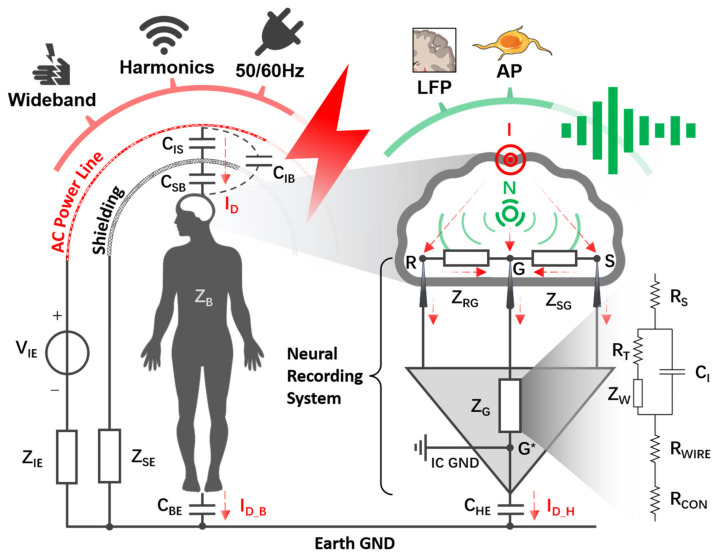
Propagation of neural signals (i.e., AP and LFP) and electromagnetic interference (e.g., 50/60 Hz electrostatic interference) from the human body to a differential recording setup. $V_{IE}$ represents the alternating electric field generated by power lines with internal resistance ($Z_{IE}$). The human body ($Z_{B}$) is capacitively coupled to the power line via $C_{IB}$ ($C_{IS}$ and $C_{SB}$, under electromagnetic shielding) and to the Earth via $C_{BE}$. $Z_{SE}$ is the sum of an electromagnetic shielding device impedance and parasitic impedance between this device and the Earth ground. Points S, R, and G, respectively, represent the positions of the signal electrode, reference electrode, and ground electrode implanted in the cortex. $Z_{SG}$ and $Z_{RG}$, respectively, represent the inter-electrode impedance from S and R to G. $Z_{G}$ represents the path impedances of the ground electrode. The path impedance is discussed in detail in the Supplementary Materials based on the Randles model. Point $G^{*}$ represents the neural recording system IC ground. $C_{HE}$ represents the coupling capacitance between the neural recording hardware system and the Earth. $I_{D\_B}$ and $I_{D\_H}$, respectively, represent displacement current through the body and neural recording hardware system, which are the dominant components of total displacement current ($I_{D}$) coupled in from the scalp. Point N (green color) represents the ideal neural signal source within the cortex, and point I (red color) represents the ideal EMI source on the brain.

The neural signal is the electric superposition of the action potential (AP: 5–500 μV, 300–10 kHz) and local field potential (LFP: <1 mV, <300 Hz) [16], which are easily plagued with various kinds of EMI aggressors, including 50/60 Hz interference from the power line, harmonic voltages from radio frequency noise, and wideband interference caused by ESD [11], as revealed in Figure 2.

Extracellular recordings from the soma suggest that the AP is initiated in the perisomatic region [17]. In contrast, LFP is believed to mainly stem from transmembrane currents resulting from the synaptic activity of neighboring dendrites within a specific volume of tissue [18]. In a three-electrode differential recording setup, the signal electrode is strategically positioned close to interested neurons, the shared reference electrode is placed at a surrounding location, and the shared ground electrode is utilized to directly connect with the neural recording system IC ground to establish a proper bias condition.

Within the cortex, the neural signal ($V_{NS\_ME\_S}$) captured by the signal microelectrode ($V_{NS\_S}$) in relation to the ground electrode ($V_{NS\_G}$) is as follows:

(1) $$V_{NS\_ME\_S}=V_{NS\_S}-V_{NS\_G}$$

Similarly, the neural signal ($V_{NS\_ME\_R}$) captured by the reference microelectrode ($V_{NS\_R}$) is as follows:

(2) $$V_{NS\_ME\_R}=V_{NS\_R}-V_{NS\_G}$$

As a consequence, the so-called neural signal captured by the microelectrodes is the differential-mode neural signal ($V_{NS\_ME\_DM}$), which is expressed as follows:

(3) $$V_{NS\_ME\_DM}=V_{NS\_ME\_S}-V_{NS\_ME\_R}=V_{NS\_S}-V_{NS\_R}$$

Power line interference is a significant source of EMI in neural signal acquisition, as the 50/60 Hz interference falls within the frequency band of neural signals ranging from 0.1 to 10 kHz. Shielding and twisting hardware leads can mitigate the capacitive and inductive coupling between the power line and the neural recording system [19]. However, the displacement current ($I_{D}$) is still coupled to the human body, as depicted in Figure 2. An alternating EMI voltage ($V_{IE}$) with internal resistance $Z_{IE}$ is utilized to simulate the alternating electric field between the power line and the Earth ground. The impedance of the human body is represented by $Z_{B}$, which is coupled to the Earth ground through capacitance $C_{BE}$. Regarding $Z_{B}$, 1000 Ω is commonly recommended for bare foot-to-hand contact in dry conditions, while values below 200 Ω may be applicable for wet conditions. This impedance range (200–1000 Ω) is also suitable for other animals [20]. Additionally, the direct coupling effect from the power line to the human body can be represented as $C_{IB}$. Consequently, the EMI of the human body ($V_{EMI\_B}$) without electromagnetic shielding can be calculated as follows:

(4) $$V_{EMI\_B}=\frac{V_{IE}\left(Z_{B}+\frac{1}{j\omega C_{BE}}\right)∕∕\left(Z_{IG^{*}}+\frac{1}{j\omega C_{HE}}\right)}{\left(Z_{B}+\frac{1}{j\omega C_{BE}}\right){∕∕\left(Z_{IG^{*}}+\frac{1}{j\omega C_{HE}}\right)+Z}_{IE}+\frac{1}{j\omega C_{IB}}}$$

where $Z_{IG^{*}}$ is the equivalent impedance from point I to point $G^{*}$. Point I represents the ideal EMI source on the brain and point $G^{*}$ represents the neural recording system IC ground. $C_{HE}$ is the coupling capacitance between the neural recording hardware system and the Earth. The equivalent circuit diagram of (4) is shown in Figure S1.

To eliminate the influence of EMI on the human body, an effective strategy is to isolate the human body from interference sources through electromagnetic shielding. Therefore, the direct coupling capacitor ($C_{IB}$) changes to the connection of $C_{IS}$, $C_{SB}$, and $C_{IB}$. Due to the weak direct coupling effect, $C_{IB}$ can be disregarded. In this case, the EMI of the human body ($V_{EMI\_\hat{B}}$) with electromagnetic shielding is as follows:

(5) $$V_{EMI\_\hat{B}}\approx \frac{V_{IE}\left(Z_{B}+\frac{1}{j\omega C_{BE}}\right)∕∕\left(Z_{IG^{*}}+\frac{1}{j\omega C_{HE}}\right)Z_{SE}}{\left[\left(Z_{B}+\frac{1}{j\omega C_{BE}}\right)∕∕\left(Z_{IG^{*}}+\frac{1}{j\omega C_{HE}}\right)+Z_{SE}+\frac{1}{j\omega C_{SB}}\right]\left(Z_{SE}+Z_{IE}+\frac{1}{j\omega C_{IS}}\right)}$$

where $Z_{SE}$ is the sum of the electromagnetic shielding device impedance and parasitic impedance between this device and the Earth ground. The Thevenin equivalent approximation of (5) can be seen in Figure S2. In (4) and (5), impedance estimation between the power line and a plane (e.g., $Z_{IE}$, $C_{IB}$, and $C_{IS}$) can be achieved based on the distributed capacitance model [21]. Meanwhile, impedance estimation between two planes (e.g., $C_{SB}$, $C_{BE}$ and $C_{HE}$) can be achieved based on the traditional parallel-plate capacitor model. However, it is often difficult to accurately estimate the capacitance between conductors in practice.

Before the neural recording hardware system is connected to the brain, the displacement current ($I_{D\_B}$) through the human body is approximately equal to $I_{D}$. After the neural recording hardware system is connected to the brain, $I_{D}$ can be expressed as follows:

(6) $$I_{D}\approx I_{D\_B}+I_{D\_H}$$

where $I_{D\_H}$ is the displacement current through the neural recording hardware system, which can also be expressed as follows:

(7) $$I_{D\_H}=\frac{V_{EMI\_B/\hat{B}}}{\frac{1}{j\omega C_{HE}}+Z_{IG^{*}}}$$

Within the cortex,

(8) $$I_{D\_H}={I_{D\_SG^{*}}+I_{D\_RG^{*}}+I}_{D\_{GG}^{*}}$$

where $I_{D\_SG^{*}}$, $I_{D\_RG^{*}}$, and $I_{D\_GG^{*}}$ are the displacement current from points S, R, and G to point $G^{*}$. Therefore, the EMIs introduced by the signal electrode ($V_{EMI\_ME\_S}$) and reference electrode ($V_{EMI\_ME\_R}$) in relation to the IC ground are as follows:

(9) $$V_{EMI\_ME\_S}=I_{D\_GG^{*}}\times Z_{G}+I_{D\_SG}\times Z_{SG}$$

(10) $$V_{EMI\_ME\_R}=I_{D\_GG^{*}}\times Z_{G}+I_{D\_RG}\times Z_{RG}$$

where $I_{D\_SG}$ and $I_{D\_RG}$ are the displacement currents from points S and R to G, respectively. $Z_{SG}$ and $Z_{RG}$ are the inter-electrode tissue impedances from S and R to G, respectively. Notably, estimating brain tissue impedance is a complex process due to various factors, including tissue type, structure, pathological state, measuring frequency, etc. As a result, estimating the impedance of the brain tissue has individual differences and dynamic variability. Currently, there is no universal formula or method to estimate the impedance of brain tissue. To guide electrode site design utilizing the impedance relationship in this work, it is assumed that the closer the distance between electrodes, the lower the impedance of brain tissue based on the traditional Ohm’s law. Significantly, the voltage reference point is with respect to the neural recording system IC ground rather than the Earth ground in (9) and (10). The equivalent circuit diagram of the two equations is shown in Figure S3. Consequently, the so-called electromagnetic interference captured by electrodes can be classified into two forms: common-mode EMI ($V_{EMI\_ME\_CM}$) and differential-mode EMI ($V_{EMI\_ME\_DM}$).

(11) $$V_{EMI\_ME\_CM}=\frac{V_{EMI\_ME\_S}+V_{EMI\_ME\_R}}{2}=Z_{G}\times I_{D\_{GG}^{*}}+\frac{I_{D\_SG}\times Z_{SG}+I_{D\_RG}\times Z_{RG}}{2}$$

(12) $$V_{EMI\_ME\_DM}=V_{EMI\_ME\_S}-V_{EMI\_ME\_R}=I_{D\_SG}\times Z_{SG}-I_{D\_RG}\times Z_{RG}$$

## 3. Neural Signal Model

**Figure 3:**
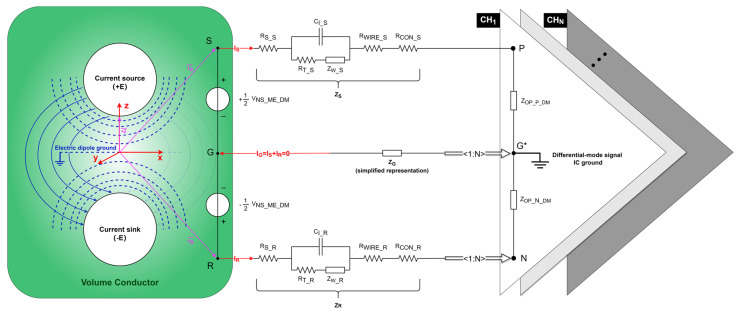
Neural signal equivalent circuit in a differential recording setup. Equipotential surfaces (blue dashed curves) and streamlines of the current (blue solid curves) for an electric dipole (white circle) consisting of the current source (+E) and current sink (-E). Each vector (purple lines) references the coordinate system (red lines). $V_{NS\_ME\_DM}$ represents the differential-mode neural signal captured from the electrolyte–electrode interface. Points S, R, and G, respectively, represent the positions of the signal electrode, reference electrode, and ground electrode implanted in the cortex. $Z_{S}$, $Z_{R}$, and $Z_{G}$ represent the path impedances of the signal, reference, and ground electrodes, respectively. Points P, N, and $G^{*}$, respectively, represent the inputs of the positive, negative, and differential-mode signal IC ground of the OPA. $I_{S}$, $I_{R}$, and $I_{G}$ represent the path current through the signal, reference, and ground electrodes, respectively. $Z_{OP\_P\_DM}$ and $Z_{OP\_N\_DM}$ represent the equivalent input differential-mode impedance of the positive and negative input of the OPA, respectively. The amplifier shares one negative and one ground input for N recording channels.

In recent years, more attention has been paid to neural modeling, particularly in neuroelectronic interface [22], spinal cord reflexes [23], neural control strategy [24], etc. Within the 1.3 L volume of the brain, the neural signal can be considered a circuit working with frequency-modulated pulses [25], which is conducted through the extracellular fluid and the surrounding tissue acting as volume conduction [26] and connected to the peripheral nervous system totaling over 150,000 km through the spinal cord [27]. Regarding the electrolyte–electrode interface, the extracellular current ($I_{EXT}$) path matches the intracellular current flow generated by differential membrane potentials [28]. In Figure 3, the active membrane region functions as a current sink (–E), whereas the inactive membrane region is a current source (+E) in relation to an electric dipole ground positioned at the bisector or infinity of the electric dipole. For the electric dipole, the blue dashed curves represent equipotential surfaces, and the blue solid curves represent the streamlines of the current. The neural signal potential produced by the electric dipole is an electric superposition based on the coordinate system with solid red lines. The pitch between the current source and the current sink is defined as 2*z* [with $\vec{+z}=\left(0,0,z\right)$, purple line]. Within the volume conductor, the extracellular current density (j) based on the origin is as follows:

(13) $$j\left(\vec{r}\right)=j_{+}\left(\vec{r}\right)+j_{-}\left(\vec{r}\right)=\frac{I_{EXT}}{4\pi }\left(\frac{\vec{r}-\vec{z}}{{\left|\vec{r}-\vec{z}\right|}^{3}}-\frac{\vec{r}+\vec{z}}{{\left|\vec{r}+\vec{z}\right|}^{3}}\right)$$

According to Ampere’s law, the voltage at a distance $\vec{r}$ from the origin is as follows:

(14) $$V\left(\vec{r}\right)=V_{+}\left(\vec{r}\right)+V_{-}\left(\vec{r}\right)=\frac{I_{EXT}\times \rho_{TIS}}{4\pi }\left(\frac{1}{\left|\vec{r}-\vec{z}\right|}-\frac{1}{\left|\vec{r}+\vec{z}\right|}\right)$$

where $\rho_{TIS}$ is the electrical resistivity of the cortical tissue. Prior research reported that grey matter impedance exhibits ohmic behavior, and its conductivity is isotropic, while white matter is ohmic, but its conductivity is direction-dependent. The electrical resistance of the cortical tissue falls within the range of 1.65–3.9 Ω·m [28]. In this work, we have assumed that the tissue resistivity around the electrodes is constant.

As depicted in Figure 3, $V_{NS\_ME\_DM}$ and $\frac{1}{2}V_{NS\_ME\_DM}$ can be expressed by the following matrix when N = 1:

(15) $$\left[\begin{array}{cc} Z_{S}+Z_{OP\_P\_DM} & {{-Z}_{R}-Z}_{OP\_N\_DM}\\ Z_{S}+Z_{OP\_P\_DM}+Z_{G} & Z_{G} \end{array}\right]\left[\begin{array}{c} I_{S}\\ I_{R} \end{array}\right]=\left[\begin{array}{c} V_{NS\_ME\_DM}\\ \frac{1}{2}V_{NS\_ME\_DM} \end{array}\right]$$

where $Z_{S}$, $Z_{R}$, and $Z_{G}$ represent the path impedances of the signal, reference, and ground electrodes, respectively. $I_{S}$ and $I_{R}$ represent the path current of the recording electrode and reference electrode. $Z_{OP\_P\_DM}$ and $Z_{OP\_N\_DM}$ represent the equivalent input differential-mode impedance of the positive and negative input of the OPA, respectively. Therefore, $I_{S}$ and $I_{R}$ are as follows:

(16) $$I_{S}=+\frac{Z_{G}\times V_{NS\_ME\_DM}+\left({Z_{R}+Z}_{OP\_N\_DM}\right)\times \frac{1}{2}V_{NS\_ME\_DM}}{\left(Z_{S}+Z_{OP\_P\_DM}\right)\times Z_{G}+\left({Z_{R}+Z}_{OP\_N\_DM}\right)\times \left(Z_{S}+Z_{OP\_P\_DM}+Z_{G}\right)}$$

(17) $$I_{R}=-\frac{Z_{G}\times V_{NS\_ME\_DM}+\left({Z_{S}+Z}_{OP\_P\_DM}\right)\times \frac{1}{2}V_{NS\_ME\_DM}}{\left(Z_{S}+Z_{OP\_P\_DM}\right)\times Z_{G}+\left({Z_{R}+Z}_{OP\_N\_DM}\right)\times \left(Z_{S}+Z_{OP\_P\_DM}+Z_{G}\right)}$$

Under full symmetry conditions (i.e., $Z_{S}=Z_{R}$ and $Z_{OP\_P\_DM}=Z_{OP\_N\_DM}$), the path current ($I_{G}$) of the ground electrode is as follows:

(18) $$I_{G}=I_{S}+I_{R}=0$$

The differential neural signal ($V_{NS\_OP\_DIFF}$) introduced by the operational amplifier (OPA) can be obtained based on the differential-mode signal IC ground.

(19) $$V_{NS\_OP\_DIFF}=I_{S}\times Z_{OP\_P\_DM}-I_{R}\times Z_{OP\_N\_DM}$$

The substitution of (16) and (17) into (19) reveals the following:

(20) $$V_{NS\_OP\_DIFF}=V_{NS\_ME\_DM}\times \frac{Z_{OP\_P\_DM}+Z_{OP\_N\_DM}}{Z_{OP\_P\_DM}+Z_{OP\_N\_DM}+Z_{S}+Z_{R}}$$

The equivalent circuit diagram of (20) is shown in Figure S4.

To record the spike, the size of the signal electrode should be equal to or smaller than that of the neuron (1–20 μm) [27]. Considering the impedance matching discussed in Section 4 later, the geometric area of the shared reference/ground electrode should be several times larger than that of the signal electrode. Hence, the signal electrode ideally captures AP and LFP signals, whereas the shared ground or reference electrode captures only LFP signals. A more complicated expression combining (3), (14), and (20) can be used to define the differential-mode neural signal.

(21) $$V_{NS\_OP\_DIFF}=\frac{1}{2}\left[\frac{I_{EXT\_AP}\times \rho_{TIS}}{4\pi }\left(\frac{1}{\left|\vec{r_{S\_AP}}-\vec{z_{AP}}\right|}-\frac{1}{\left|\vec{r_{S\_AP}}+\vec{z_{AP}}\right|}\right)+\frac{I_{EXT\_LFP}\times \rho_{TIS}}{4\pi }\left(\frac{1}{\left|\vec{r_{S\_LFP}}-\vec{z_{LFP}}\right|}-\frac{1}{\left|\vec{r_{S\_LFP}}+\vec{z_{LFP}}\right|}\right)\right.\;\left.-\frac{I_{EXT\_LFP}\times \rho_{TIS}}{4\pi }\left(\frac{1}{\left|\vec{r_{R\_LFP}}-\vec{z_{LFP}}\right|}-\frac{1}{\left|\vec{r_{R\_LFP}}+\vec{z_{LFP}}\right|}\right)\right]\times \left(\frac{Z_{OP\_P\_DM}+Z_{OP\_N\_DM}}{Z_{OP\_P\_DM}+Z_{OP\_N\_DM}+Z_{S}+Z_{R}}\right)$$

where $I_{EXT\_AP}$ represents the extracellular current resulting from the AP firing of single cortical neurons around the signal electrodes. $I_{EXT\_LFP}$ represents the extracellular current resulting from the LFP firing of neighboring dendrites around the signal and reference electrodes. $\vec{r_{S\_AP}}$ represents the distance vector from the signal electrodes to the origin in the AP recording. $\vec{r_{S\_LFP}}$ and $\vec{r_{R\_LFP}}$, respectively, represent the distance vectors from the signal and reference electrodes to the origin in the LFP recording. $\vec{{\pm z}_{AP}}$ represents the distance vector from the AP current source (+) or sink (–) to the origin. $\vec{{\pm z}_{LFP}}$ represents the distance vector from the LFP current source (+) or sink (–) to the origin.

According to the first term of (21), controlling various extracellular currents and the distances between the electrode and current source is difficult due to the uncertainty in the nerve discharge intensity and current flow direction. To obtain a greater AP value recorded by the electrodes, the signal electrode should be as close as possible to the neuron of interest (i.e., AP current sink), that is, to minimize the value of $\left|\vec{r_{S\_AP}}+\vec{z_{AP}}\right|$. To achieve high-resolution single-unit activity (SUA) recordings, it is recommended to place the signal electrode within 20 μm of the neuron body [29]. Others have argued for an even larger range of 50–100 µm [4]. Additionally, although multi-unit activity (MUA) typically yields a relatively lower signal-to-noise ratio (SNR) than SUA, it is worth capturing within a radius in the range of 100–150 µm [4]. Nevertheless, an alternative opinion suggests that the ideal distance for MUA recording is around 140–300 µm [30]. Any other signals beyond these specified ranges were considered noise. To avoid weakening neural signals caused by the close distance between electrodes, a practical approach is to ensure that the distance between any two electrodes in recording, reference, and ground electrode is over 20 µm based on (1), (2) and (3). Meanwhile, to maximize the magnitude of the LFP, the distance ($\left|\vec{r_{S\_LFP}}+\vec{z_{LFP}}\right|$) between the signal electrode and the LFP current sink should be minimized, whereas the distance ($\left|\vec{r_{R\_LFP}}+\vec{z_{LFP}}\right|$) between the reference electrode and the LFP current sink should be maximized. However, it is impractical to predict the position of the LFP current sink before electrode implantation. Accordingly, a practical approach is to maximize the distance between the signal and the reference electrode within the influence range of the LFP. There are various opinions on the location of LFP, with lateral spreads of 200–400 µm [31,32], 600–1000 µm [33], 2–3 mm [34,35], and 5 mm [36], and a vertical spread on the scale of centimeters [37].

According to the second term of (21), it is necessary to make the equivalent input differential-mode impedance of the positive ($Z_{OP\_P\_DM}$) and negative ($Z_{OP\_N\_DM}$) inputs of the OPA as large as possible and the path impedance of the signal electrode ($Z_{S}$) and reference electrode ($Z_{R}$) as small as possible. As mentioned in the Supplementary Materials, the equivalent input impedance (at 1 kHz) of OPA ranges from 6.6 MΩ (20 pF) [38] to 14.9 MΩ (9.7 pF) [39], and the impedance of the commercial probe (at 1 kHz) ranges from 5 kΩ to 2.5 MΩ [7]. The attenuation ratio of the neural signal from the electrode to the OPA input is ~0.03%–27.38%. Furthermore, the subsequent amplifier stages comprising, one or more cascaded amplifier configurations, offer an additional gain range of 33–63 dB [40,41,42]. The gain selection is mainly influenced by the specific application requirements and characteristics of the power supply.

## 4. Common-Mode EMI Model

**Figure 4:**
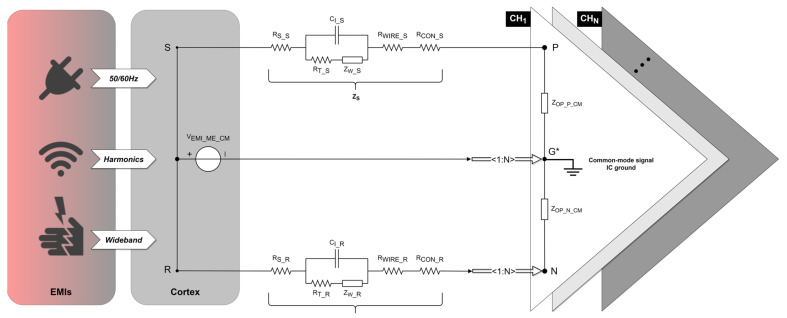
Common-mode interference equivalent circuit in a differential recording setup. The EMI source (left rectangle) aggresses the cortex (right rectangle) in a common-mode form. $V_{EMI\_ME\_CM}$ represents the common-mode interference voltage introduced from the electrolyte–electrode interface based on the common-mode signal IC ground. Points S and R, respectively, represent the positions of the signal electrode and reference electrode implanted in the cortex. $Z_{S}$ and $Z_{R}$ represent the path impedances of the signal and reference electrode, respectively. Points P, N, and $G^{*}$, respectively, represent the inputs of the positive, negative, and common-mode signal IC ground of the OPA. $Z_{OP\_P\_CM}$ and $Z_{OP\_N\_CM}$ represent the equivalent input common-mode impedance of the positive and negative input of the OPA, respectively. The amplifier shares one negative and one ground input for N recording channels.

Common-mode interference has been extensively studied, yet its quantification methods primarily focus on electrode impedance and IC design while neglecting the impact of electrode positioning at the electrolyte–electrode interface. In Figure 4, the CMI introduced by the positive ($V_{CMI\_OP\_P}$) and negative ($V_{CMI\_OP\_N}$) inputs of the OPA can be obtained based on the common-mode signal IC ground.

(22) $$V_{CMI\_OP\_P}=V_{EMI\_ME\_CM}\times \frac{Z_{OP\_P\_CM}}{Z_{OP\_P\_CM}+Z_{S}}$$

(23) $$V_{CMI\_OP\_N}=V_{EMI\_ME\_CM}\times \frac{Z_{OP\_N\_CM}}{Z_{OP\_N\_CM}+Z_{R}}$$

According to (11), the differential CMI introduced by the OPA ($V_{CMI\_OP\_DIFF}$) can be obtained.

(24) $$V_{CMI\_OP\_DIFF}=\left(Z_{G}\times I_{D\_G^{*}G}+\frac{I_{D\_SG}\times Z_{SG}+I_{D\_RG}\times Z_{RG}}{2}\right)\times \left(\frac{Z_{OP\_P\_CM}}{Z_{OP\_P\_CM}+Z_{S}}-\frac{Z_{OP\_N\_CM}}{Z_{OP\_N\_CM}+Z_{R}}\right)$$

According to the first term (i.e., $V_{EMI\_ME\_CM}$) of (24), First, $Z_{G}$ should be reduced. Accordingly, the contribution of $I_{D\_H}$ is approximately only $I_{D\_{GG}^{*}}$ mentioned in (8).

(25) $${I_{D\_H}\approx I}_{D\_{GG}^{*}}$$

The substitution of (7) and (25) into (24) reveals the following:

(26) $$V_{CMI\_OP\_DIFF}=\left(Z_{G}\times \frac{V_{EMI\_B/\hat{B}}}{\frac{1}{j\omega C_{HE}}+Z_{IG^{*}}}+\frac{I_{D\_SG}\times Z_{SG}+I_{D\_RG}\times Z_{RG}}{2}\right)\times \left(\frac{Z_{OP\_P\_CM}}{Z_{OP\_P\_CM}+Z_{S}}-\frac{Z_{OP\_N\_CM}}{Z_{OP\_N\_CM}+Z_{R}}\right)$$

Second, minimizing $\frac{V_{EMI\_B/\hat{B}}}{1/j\omega C_{HE}+Z_{IG^{*}}}$ (i.e., $I_{D\_GG^{*}}$) is beneficial for reducing $V_{CMI\_OP\_DIFF}$. According to (4) and (5), it is not easy to control $V_{IE}$, $Z_{IE}$, $C_{IB}$, $C_{IS}$, and $Z_{IG^{*}}$, given the unpredictability and superposition of EMI sources. Additionally, once the tested organism is selected, $Z_{B}$ is not controlled artificially. A straightforward strategy is the minimization of $C_{HE}$ and $C_{BE}$. From a physical length perspective, the distance between the neural recording hardware system/the human body and the Earth ground should be as large as possible. Under an electromagnetic shield, using shielding materials with excellent electrical conductivity and ensuring good grounding of the shielding can effectively mitigate $Z_{SE}$. Additionally, $C_{SB}$ should be minimized. A common mistake is to place shielding devices too close to the body.

Third, because $I_{D\_SG}$ and $I_{D\_RG}$ are both components of $I_{D\_GG^{*}}$, the above optimization measures for reducing $I_{D\_{GG}^{*}}$ also help reduce $I_{D\_SG}$ and $I_{D\_RG}$. One common oversight is ignoring the distance between the signal/reference electrode site and ground electrode site, thus accidentally creating a high impedance $Z_{SG}$ and/or $Z_{RG}$. Notably, as the path impedance of the signal electrode decreases to the order of kΩ, the path impedance of the ground electrode decreases significantly with an increase in the geometric area or effective surface area. Consequently, $Z_{SG}$ and $Z_{RG}$ must not be ignored. A rough estimation of the literature suggests that the CMI induced between the signal electrode and ground electrode is an order of magnitude of 1 mV when the ground electrode is positioned in the cortex. However, if the ground electrode is located elsewhere in the head, this voltage may increase to an order of magnitude of 10 mV. When the ground electrode is placed on the chest, a higher CMI of an order of magnitude of 100 mV can be observed [5,43,44]. Therefore, the optimal implantation sites of the ground electrode should be in the cortex and close to the signal/reference electrode.

According to the second term of (24), its value minimization measure yields two impedance matching schemes: rigorous impedance matching and extreme impedance matching. For a multiple-channel recording setup, $Z_{OP\_N\_CM}$ is calculated as follows:

(27) $$Z_{OP\_N\_CM}=\frac{Z_{OP\_P\_CM}}{N}$$

where N represents the number of recording channels. Based on rigorous impedance matching, the path impedance must be matched to (27).

(28) $$Z_{R}=\frac{Z_{S}}{N}$$

The value of $V_{CMI\_OP\_DIFF}$ in (24) can be obtained as follows:

(29) $${V_{CMI\_OP\_DIFF}=V}_{CMI\_ME\_CM}\times \left(\frac{Z_{OP\_P\_CM}}{Z_{OP\_P\_CM}+Z_{S}}-\frac{\frac{Z_{OP\_P\_CM}}{N}}{\frac{Z_{OP\_P\_CM}}{N}+\frac{Z_{S}}{N}}\right)=0$$

However, achieving rigorous impedance matching is challenging due to manufacturing process errors and the unpredictability of electrolyte–electrode interface impedance for passive neural probes. Therefore, extreme impedance matching emerged.

To protect neural signals, as mentioned in (21), a simple and extreme method is that both $\frac{Z_{OP\_P\_CM}}{Z_{OP\_P\_CM}+Z_{S}}$ and $\frac{Z_{OP\_N\_CM}}{Z_{OP\_N\_CM}+Z_{R}}$ approach one rather than zero. Specifically, $Z_{OP\_P\_CM}$ and $Z_{OP\_N\_CM}$ should be as large as possible while minimizing $Z_{S}$ and $Z_{R}$ as much as possible.

(30) $$Z_{S}/Z_{R}\ll Z_{OP\_N\_CM}$$

Based on extreme impedance matching, the value of $V_{CMI\_OP\_DIFF}$ can be obtained as follows:

(31) $${V_{CMI\_OP\_DIFF}\approx V}_{CMI\_ME\_CM}\times \left(\frac{Z_{OP\_P\_CM}}{Z_{OP\_P\_CM}}-\frac{Z_{OP\_N\_CM}}{Z_{OP\_N\_CM}}\right)=0$$

Although this scheme is simple and feasible for impedance matching, it poses a significant risk for subsequent amplifier stages, particularly the CMI range design. The CMI captured by the electrode enters the amplifier input almost without attenuation. In the worst-case scenario, an excessive CMI can cause saturation of the analog front-end (AFE), leading to a complete failure of the neural recording system. As a result, extreme impedance matching must cooperate with the minimization measure of $V_{CMI\_ME\_CM}$ mentioned in the first term of (24).

The magnitude of the CMI to the final obtained neural signal is closely linked to the CMRR index of the OPA. The main source of CMI can reach up to 100 mV_pp_ due to the capacitive coupling of the mains. The amplifier’s CMRR depends on the on-chip device mismatch and typically needs to be greater than 70 dB [45] for a detectable neural signal as low as 5 µV_rms_. Therefore, a high CMRR is an important characteristic of neural amplifiers.

## 5. Differential-Mode EMI Model

**Figure 5:**
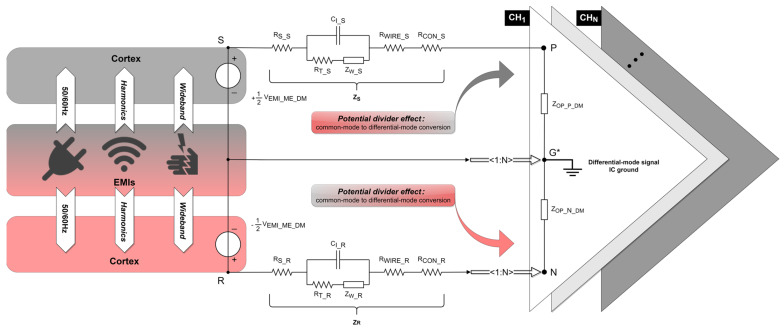
Differential-mode interference equivalent circuit in a differential recording setup. The EMI source (middle rectangle) aggresses the cortex (upper/bottom rectangle) in a differential-mode form. $V_{EMI\_ME\_DM}$ represents the differential-mode interference voltage introduced from the electrolyte–electrode interface based on the differential-mode signal IC ground. Points S and R, respectively, represent the positions of the signal electrode and reference electrode implanted in the cortex. $Z_{S}$ and $Z_{R}$ represent the path impedances of the signal and reference electrode, respectively. Points P, N, and $G^{*}$, respectively, represent the inputs of the positive, negative, and differential-mode signal IC ground of the OPA. $Z_{OP\_P\_DM}$ and $Z_{OP\_N\_DM}$ represent the equivalent input differential-mode impedance of the positive and negative input of the OPA, respectively. The amplifier shares one negative and one ground input for N recording channels.

The differential-mode signal at the amplifier input would ideally contain only the neural signal sensed by the electrodes. However, due to the oversight of electrode design, as well as the impedance mismatch between the shared reference input and each signal input, the OPA introduces differential-mode interference ($V_{DMI\_OP\_DIFF}$), as depicted in Figure 5.

(32) $$V_{DMI\_OP\_DIFF}=\left(V_{EMI\_ME\_DM}\times \frac{Z_{OP\_P\_DM}+Z_{OP\_N\_DM}}{Z_{OP\_P\_DM}+Z_{OP\_N\_DM}+Z_{S}+Z_{R}}\right)+\left[V_{EMI\_ME\_CM}\times ∆\left(\frac{Z_{OP\_P\_CM}}{Z_{OP\_P\_CM}+Z_{S}}-\frac{Z_{OP\_N\_CM}}{Z_{OP\_N\_CM}+Z_{R}}\right)\right]$$

In a multiple-channel differential recording setup with one shared reference input, any common-mode perturbations introduced from the electrolyte–electrode interface must result in input-referenced differential-mode interference (DMI) of the neural amplifier due to imbalances between electrode path impedances and/or equivalent input common-mode impedances of the OPA. This phenomenon is also called the potential divider effect [19]. Substituting (11) and (12) into (32) yields the following:

(33) $$\begin{array}{ll} V_{DMI\_OP\_DIFF} & =\left[\left(I_{D\_SG}\times Z_{SG}-I_{D\_RG}\times Z_{RG}\right)\times \left.\frac{Z_{OP\_P\_DM}+Z_{OP\_N\_DM}}{Z_{OP\_P\_DM}+Z_{OP\_N\_DM}+Z_{S}+Z_{R}}\right]\right.\\ & +\left[\left(I_{D\_G^{*}G}\times Z_{G}+\frac{I_{D\_SG}\times Z_{SG}+I_{D\_RG}\times Z_{RG}}{2}\right)\right.\left.\times \Delta \left(\frac{Z_{OP\_P\_CM}}{Z_{OP\_P\_CM}+Z_{S}}-\frac{Z_{OP\_N\_CM}}{Z_{OP\_N\_CM}+Z_{R}}\right)\right] \end{array}$$

According to the first term of (33), reducing $\frac{Z_{OP\_P\_DM}+Z_{OP\_N\_DM}}{Z_{OP\_P\_DM}+Z_{OP\_N\_DM}+Z_{S}+Z_{R}}$ is unfeasible, as it would attenuate the neural signal, as analyzed in (21). $\left(I_{D\_SG}\times Z_{SG}-I_{D\_RG}\times Z_{RG}\right)$ should be as small as possible. This requires full consideration of the relative positions of signal/reference/ground electrode sites when designing a neural probe. In the current component of $\left(I_{D\_SG}\times Z_{SG}-I_{D\_RG}\times Z_{RG}\right)$, it is challenging to control the magnitude of $I_{D\_SG}$ or $I_{D\_RG}$ given the unpredictability and superposition of the EMI sources, efforts can be made to ensure $I_{D\_SG}\approx I_{D\_RG}$. A practical approach involves placing the signal electrode close to the reference electrode. However, such settings must be extremely careful because a close distance between the signal electrode and reference electrode may weaken the LFP component, even the AP component, according to Section 3. In the impedance component of $\left(I_{D\_SG}\times Z_{SG}-I_{D\_RG}\times Z_{RG}\right)$, the ideal position for the ground electrode is the midpoint between the signal electrode and reference electrode to ensure $Z_{SG}\approx Z_{RG}$.

According to the second term of (33), the significance of minimizing CMI from the electrolyte–electrode interface is not only beneficial for the subsequent implementation of common-mode suppression on ASIC but also for reducing the conversion of CMI into DMI due to impedance mismatch.

## 6. In Vivo Experiments

Redesigning the neural probe is necessary to verify the equivalent circuit model. As depicted in Figure S6, the recording electrode is typically circular in shape with a diameter of less than 20 µm. To ensure impedance matching, the geometric area of the reference electrodes is N times greater than that of the recording electrode. Additionally, the ground electrode should be strategically designed in the cortical region with a larger geometric area for optimal performance. Regarding the configuration of the electrodes at the electrolyte–electrode interface, the inter-electrode distance between any two types of electrodes in recording, reference, and ground electrodes should be >20 µm (Maximum neuron size) to avoid weakening AP magnitude caused by the close distance, and <200 µm (minimum LFP local) to locate in the LFP spread range as discussed in (21). Notably, the smaller the inter-electrode distance, the smaller the LFP magnitude, but the smaller the common-mode EMI magnitude as mentioned in (24). In addition, positioning the ground electrode at the midpoint between the signal and reference electrodes is necessary to minimize the differential-mode EMI magnitude as discussed in (33). The detailed optimization design of the electrodes in a multiple-channel recording system is available in the Supplementary Materials.

**Figure 6:**
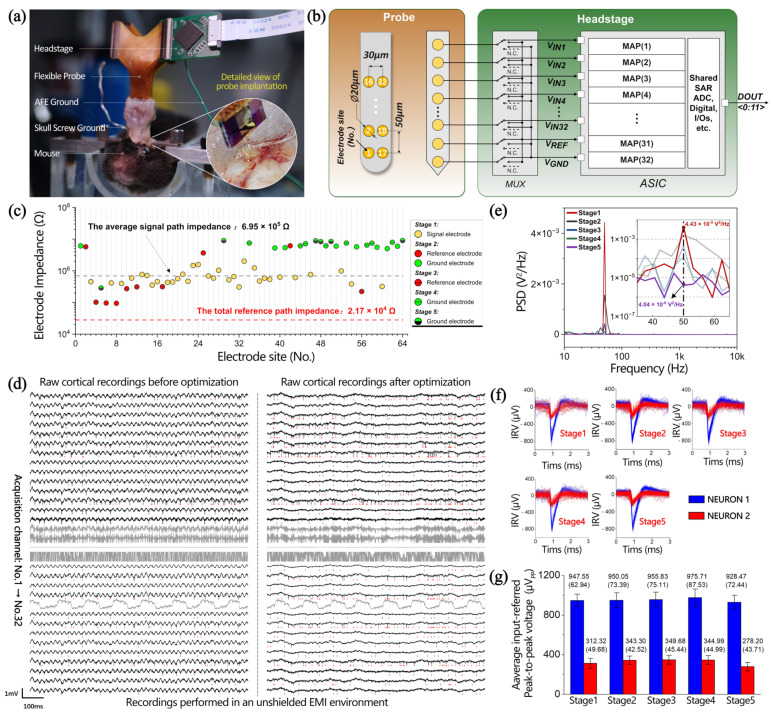
(**a**) In vivo experiment recording setup and a detailed view of the flexible probe implantation. (**b**) System architecture of the configurable 32-channel neural recording system prototype. Left: schematic of electrode configuration in a single shank. Right: schematics of the circuit architecture for the headstage. MAPs, modular analog pixels. (**c**) Electrode impedance at 1 kHz. (**d**) Raw cortical recordings before and after optimization in an unshielded EMI environment. Red marks represent the time point of the neuron firing. Taking the acquisition results of No.7 as an example, (**e**) power spectral density (PSD) of raw cortical recordings, (**f**) input-referred voltage (IRV) of spike, and (**g**) input-referred peak-to-peak voltage of spike and standard deviation statistics are obtained in five stages.

To popularize the model on the unoptimized probe, a configurable neural recording system for commercial electrodes [46] was adopted, which enhanced EMI rejection performance for neural signal recordings. As revealed in Figure 6a, the fabricated experimental setup was used, in which a flexible neural probe connected to the headstage using a zero-insertion force connector was used to record neural signals from the posterior motor cortex and cingulate cortex areas of an awake mouse. All the experimental procedures were approved by the Institutional Animal Care and Use Committee of the Institute of Neuroscience, Center for Excellence in Brain Science and Intelligence Technology, Chinese Academy of Sciences. All in vivo experiments were performed in an unshielded experiment environment. The flexible probe consists of four shanks (inset of Figure 6a), each equipped with 32 electrodes measuring 20 μm in diameter. The electrode site pitch is 30 μm horizontally and 50 μm vertically, and its site numbers are displayed on the left side of Figure 6b. Two out of the four shanks were chosen as recording channels to build the configurable 32-channel neural recording system shown on the right side of Figure 6b. In the headstage, the type of signal input from the electrode is categorized as either signal, reference, or ground using a time-division multiplexer (MUX). Following the MUX, there are 32 modular analog pixels (MAPs) and a shared 12-bit successive approximation register (SAR) ADC to generate a stable digital signal stream. Subsequently, a digital control block is deployed to achieve data transmission via a 12-bit digital bus DOUT<0:11> at a rate of 1.024 MSPS. The flexible probe had been previously implanted in the cortical region of the mouse for more than 12 months before recording. Figure 6c displays the fresh impedances of the 64 electrodes. To ensure the uniformity of impedance matching, 32 channels with relatively consistent electrode impedances were selected as the signal electrode, as indicated by the yellow points. The impedance of the signal electrode is primarily capacitive, and the average impedance magnitude measured (at 1 kHz) is approximately 6.95 × 10^5^ Ω. Meanwhile, the input capacitance of the OPA is 21 pF. Thus, its equivalent input impedance (at 1 kHz) is 7.6 MΩ. Here, the main strategies to enhance the EMI rejection performance for the configurable 32-channel neural recording system prototype were as follows:

(1) Maximize the number of ground electrodes implanted in the cortex to reduce the inter-electrode impedance of $Z_{SG}$ and/or $Z_{RG}$ and the path impedance of $Z_{G}$, thereby together reducing the introduction of the CMI from the electrolyte–electrode interface.
(2) Optimize the number of reference electrodes implanted in the cortex to achieve impedance matching, thereby avoiding the introduction of DMI due to the potential divider effect.
(3) Place the positions of the signal, reference, and ground electrode in a staggered manner in the cortex to roughly approach the equal trend of $I_{D\_SG}$ and $I_{D\_RG}$ or $Z_{SG}$ and $Z_{RG}$, thereby reducing the introduction of DMI from the electrolyte–electrode interface. These optimization strategies are divided into five progressive stages. The raw cortical recording results before and after optimization are displayed in Figure 6d.

In the first stage (i.e., the initial stage before optimization), the recording setup was configured with 32 OPA-positive inputs connected to 32 selected signal electrodes as well as a shared OPA-negative input and the OPA-ground input linked together to the skull ground. Notably, the skull ground mentioned here is achieved by contacting a silver wire with a diameter of 300 μm with cerebrospinal fluid. Considering the cross-sectional area of the silver wire as the interface contact area, the path impedance of the ground electrode is at least two orders of magnitude lower than that of the signal electrode, approximately in the kiloohm range. Raw cortical recordings show observable 50 Hz interference from the mains, with a PSD of 4.43 × 10^−3^ V^2^/Hz (red line in Figure 6e). As depicted in Figure 6f, a time-aligned analysis of raw cortical recordings reveals that the neural recording system successfully captured the spiking activities of two active neurons: Neuron1 and Neuron2. During the first stage, Neuron1 demonstrates an average input-referred peak-to-peak voltage of 947.55 μV_pp_ with a standard deviation of 62.94. In comparison, Neuron2 exhibits a lower average input-referred peak-to-peak voltage of 312.32 μV_pp_ along with a standard deviation of 49.68, as illustrated in Figure 6g.

In the second stage, the OPA negative input was removed from the skull ground and connected to four reference electrodes with a parallel impedance of 4.67 × 10^4^ Ω (at 1 kHz). The OPA ground electrode continues to be connected to the skull ground, while eight ground electrodes were added with a parallel impedance of 7.01 × 10^5^ Ω (at 1 kHz). The PSD of the 50 Hz interference is 1.56 × 10^−3^ V^2^/Hz (black line in Figure 6e). Compared to the first stage, the 50 Hz interference contribution is reduced by approximately three times; however, the changes in input-referred peak-to-peak spike voltage of Neuron1 and Neuron2 are very small, with peak-to-peak values of 950.05 μV_pp_ (Neuron1) and 343.30 μV_pp_ (Neuron2) and standard deviations of 73.39 (Neuron1) and 42.52 (Neuron2).

In the third stage, six reference electrodes were added based on the second stage, yielding a total reference path impedance of 2.17 × 10^4^ Ω (at 1 kHz), which closely matches the 1:32 ratio to the impedance of the signal electrode. Compared to the first stage, the PSD of the 50 Hz interference was reduced by approximately ten times, measuring at 4.30 × 10^−4^ V^2^/Hz (blue line in Figure 6e). However, the changes in input-referred peak-to-peak spike voltage of Neuron1 and Neuron2 are very small, with peak-to-peak values of 955.83 μV_pp_ (Neuron1) and 349.68 μV_pp_ (Neuron2) and standard deviations of 75.11 (Neuron1) and 45.44 (Neuron2).

In the fourth stage, eight ground electrodes were added based on the third stage, introducing a parallel ground impedance of 8.73 × 10^5^ Ω (at 1 kHz). Compared to the first stage, this led to a 39 times reduction in the PSD of 50 Hz interference, down to 1.15 × 10^−4^ V^2^/Hz (green line in Figure 6e). Compared with the previous three stages, the changes in the input-referred peak-to-peak spike voltage of the two neurons are very small.

In the fifth stage (i.e., the final stage after optimization), six ground electrodes were added based on the fourth stage, bringing the total number of ground electrodes to 22. This leads to the introduction of a parallel ground path impedance of 1.51 × 10^5^ Ω based on the skull ground impedance (kΩ order of magnitude). Although such an introduction has little effect on the change in the path impedance of the ground electrode, the interference is greatly reduced due to the change in inter-electrode impedance ($Z_{SG}$ and/or $Z_{RG}$). Compared with the 50 Hz interference before optimization, the optimized results have no obvious interference, as revealed in Figure 6d. Compared to the first stage, the PSD of the 50 Hz interference decreased by 1096 times and remained at 4.04 × 10^−6^ V^2^/Hz. Although there is a slight decrease in the input-referred peak-to-peak spike voltage of the two neurons, there is still no significant attenuation considered due to the reasonable fluctuations within the standard deviation.

In summary, effective EMI rejection has been achieved in in vivo experiments through optimization measures, including (1) maximizing the number of ground electrodes, (2) optimizing the number/position of reference electrodes, and (3) arranging the positions of the signal, reference, and ground electrodes in a staggered manner. Comparing before and after optimization, the PSD of the 50 Hz interference decreased by three orders of magnitude without significant changes in the input-referred peak-to-peak spike voltages of the two neurons.

## 7. Conclusions

An equivalent circuit model was developed to maximize the magnitude of the neural signal and minimize the influence of EMI from the electrolyte–electrode interface to the amplifier input. For the configuration of the electrodes implanted in the cortex, some design guidelines should be followed to enhance the EMI rejection performance for a multiple-channel neural recording system:

(1) The space between any two electrodes in the recording, reference, and ground electrode should be >20 µm to avoid accidentally reducing the magnitude of the AP.
(2) On the basis of guideline 1, the space between the signal and reference electrode should be 20–200 µm (minimum LFP local) to meet the different requirements of the magnitude of LFP captured by electrodes and DMI from the electrolyte–electrode interface.
(3) On the basis of guideline 1, place the ground electrode at the midpoint of the signal and reference electrodes in the cortex to alleviate the influence of both CMI and DMI from the electrolyte–electrode interface.

For the path impedance of electrodes and the equivalent input impedance of OPA, other design guidelines should be followed to enhance the EMI rejection performance in a multiple-channel neural recording system:

(4) Minimize the path impedance of the ground electrode to reduce the introduction of CMI from the electrolyte–electrode interface and DMI due to the potential divider effect.
(5) On the basis of guideline 4, minimize the path impedance of the signal and reference electrode and maximize the equivalent input differential-mode impedance of OPA to enhance the magnitude of neural signals.
(6) On the basis of guideline 4, match the path impedance of the signal and reference electrode with the equivalent input common-mode impedance of OPA to enhance the common-mode rejection performance of the overall recording system and reduce the introduction of DMI due to the potential divider effect.

This equivalent circuit model was validated by an in vivo experiment using a configurable 32-channel neural recording system. As a result, the PSD of the 50 Hz interference decreased by three orders of magnitude without a significant reduction in the input-referred peak-to-peak spike voltage of two neurons.

## Data Availability

Data are contained within the article.

## Supplementary Materials

The following supporting information can be downloaded at https://www.mdpi.com/article/10.3390/bios14070343/s1 [6,7,38,39,41,42,47,48,49,50,51,52,53,54,55,56,57,58,59,60,61,62]. Figure S1. The EMI of the human body without electromagnetic shielding (Equation (S4)); Figure S2. The EMI of the human body with electromagnetic shielding Equation (5); Figure S3. The EMI of the signal electrode and reference electrode in relation to the IC ground Equations (11) and (12); Figure S4. The differential neural signal introduced by the operational amplifier Equation (22); Figure S5: The impedance range varies for each part of the signal electrode’s impedance path; Figure S6: Optimal design of the electrodes in a multiple-channel neural recording system. S, signal electrode; R, reference electrode; G, ground electrode.
