# Supplementary material for: Modeling and Analysis of Environmental Electromagnetic Interference in Multiple-Channel Neural Recording Systems for High Common-Mode Interference Rejection Performance
Source: Biosensors (Basel). 2024 Jul 15;14(7):343. doi: 10.3390/bios14070343 (PMC11275126; doi:10.3390/bios14070343)
Supplement: Supplementary file 1 [file biosensors-14-00343-s001.zip › biosensors-3077256-supplementary.pdf]

Supplementary

Modeling and Analysis of Environmental Electromagnetic Interference in Multiple-Channel Neural Recording Systems for High Common-Mode Interference Rejection Performance

1. Equivalent circuit diagram

To clarify the entire system pictured in the manuscript, simplified equivalent circuit diagrams are added to clearly describe the specific equation.

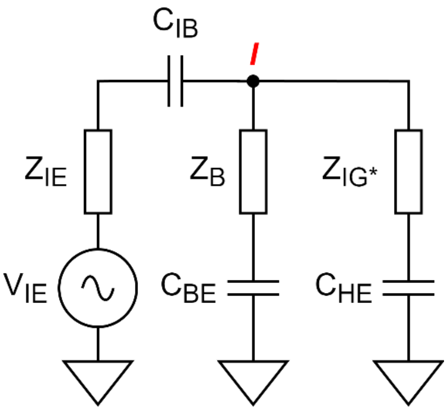

Figure S1. The EMI of the human body without electromagnetic shielding (Equation S4).

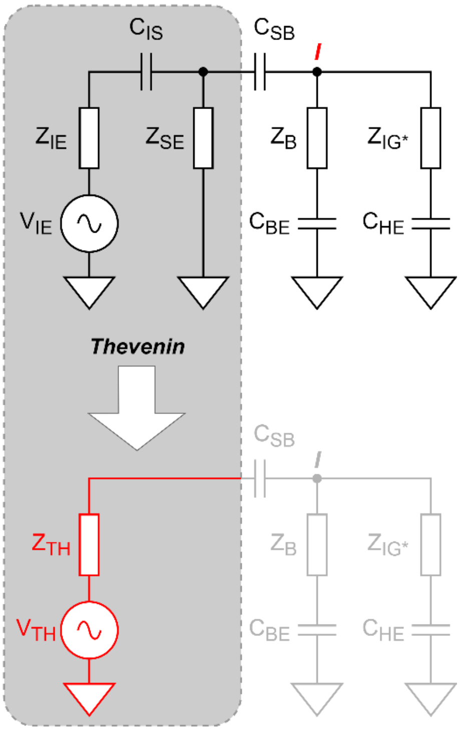

Figure S2. The EMI of the human body with electromagnetic shielding (Equation S5).

Based on Thevenin's theorem,

$$V_{EMI\_\hat{B}} = \frac{V_{TH}\left(Z_B + \frac{1}{j\omega C_{BE}}\right) // \left(Z_{IG^*} + \frac{1}{j\omega C_{HE}}\right)}{\left[\left(Z_B + \frac{1}{j\omega C_{BE}}\right) // \left(Z_{IG^*} + \frac{1}{j\omega C_{HE}}\right) + \frac{1}{j\omega C_{SB}} + Z_{TH}\right]} \tag{S1}$$

And,

$$V_{TH} = \frac{Z_{SE}}{\left(Z_{SE} + Z_{IE} + \frac{1}{j\omega C_{IS}}\right)} \tag{S2}$$

And,

$$Z_{TH} = Z_{SE} // \left(Z_{IE} + \frac{1}{j\omega C_{IS}}\right) \approx Z_{SE} \tag{S3}$$

The Z_{SE} is very small compared to $\left(Z_{IE} + \frac{1}{j\omega C_{IS}}\right)$ due to shielding materials with excellent electrical conductivity and ensuring good grounding. Therefore, Z_{TH} simplifies to Z_{SE}. Therefore, the EMI of the human body with electromagnetic shielding can be obtained.

$$V_{EMI\_\hat{B}} \approx \frac{V_{IE}\left(Z_B + \frac{1}{j\omega C_{BE}}\right) // \left(Z_{IG^*} + \frac{1}{j\omega C_{HE}}\right) Z_{SE}}{\left[\left(Z_B + \frac{1}{j\omega C_{BE}}\right) // \left(Z_{IG^*} + \frac{1}{j\omega C_{HE}}\right) + Z_{SE} + \frac{1}{j\omega C_{SB}}\right]\left(Z_{SE} + Z_{IE} + \frac{1}{j\omega C_{IS}}\right)} \tag{S4}$$

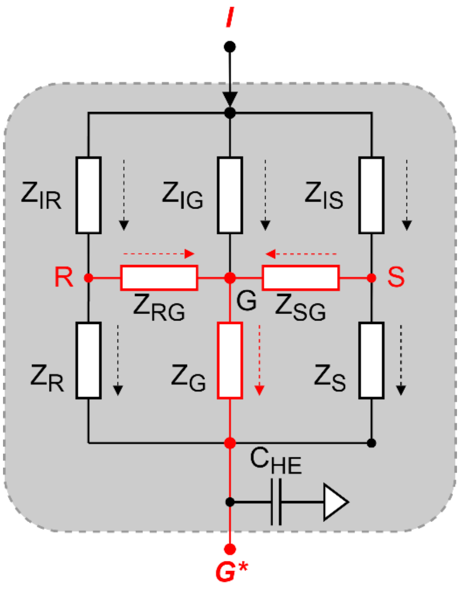

Figure S3. The EMI of the signal electrode and reference electrode in relation to the IC ground (Equations S11 and S12).

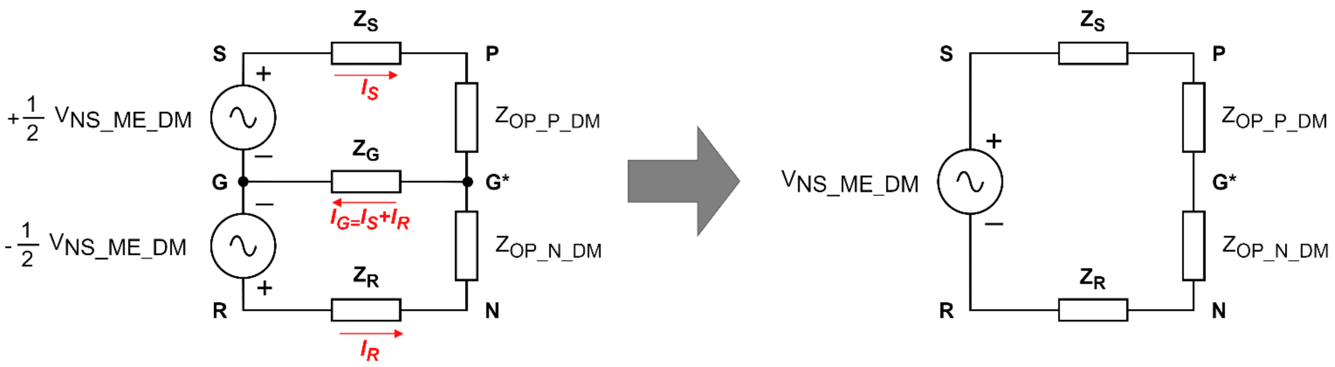

Figure S4. The differential neural signal introduced by the operational amplifier (Equation S22).

Based on the differential-mode signal IC ground,

$$V_{NS\_ME\_DM} = V_S - V_R = I_S \times (Z_S + Z_{OP\_P\_DM}) - I_R \times (Z_R + Z_{OP\_N\_DM}) \tag{S5}$$

$$\begin{aligned} +\frac{1}{2} V_{NS\_ME\_DM} &= I_S \times (Z_S + Z_{OP\_P\_DM}) + I_G \times Z_G \\ &= I_S \times (Z_S + Z_{OP\_P\_DM} + Z_G) + I_R \times Z_G \end{aligned} \tag{S6}$$

The above formula is expressed by the matrix as follows.

$$\begin{bmatrix} Z_S + Z_{OP\_P\_DM} & -Z_R - Z_{OP\_N\_DM} \\ Z_S + Z_{OP\_P\_DM} + Z_G & Z_G \end{bmatrix} \begin{bmatrix} I_S \\ I_R \end{bmatrix} = \begin{bmatrix} V_{NS\_ME\_DM} \\ 1/2\, V_{NS\_ME\_DM} \end{bmatrix} \tag{S7}$$

Thus,

$$\begin{bmatrix} V_{NS\_ME\_DM} \\ 1/2\, V_{NS\_ME\_DM} \end{bmatrix} \begin{bmatrix} Z_S + Z_{OP\_P\_DM} & -Z_R - Z_{OP\_N\_DM} \\ Z_S + Z_{OP\_P\_DM} + Z_G & Z_G \end{bmatrix}^{-1} = \begin{bmatrix} I_S \\ I_R \end{bmatrix} \tag{S8}$$

Further,

$$\frac{1}{(Z_S + Z_{OP\_P\_DM}) \times Z_G + (Z_R + Z_{OP\_N\_DM}) \times (Z_S + Z_{OP\_P\_DM} + Z_G)} \begin{bmatrix} Z_G & Z_R + Z_{(} \\ -Z_S - Z_{OP\_P\_DM} - Z_G & Z_S + Z_{(} \end{bmatrix} = \begin{bmatrix} I_S \\ I_R \end{bmatrix} \tag{S9}$$

Therefore,

$$I_S = +\frac{Z_G \times V_{NS\_ME\_DM} + (Z_R + Z_{OP\_N\_DM}) \times 1/2\, V_{NS\_ME\_DM}}{(Z_S + Z_{OP\_P\_DM}) \times Z_G + (Z_R + Z_{OP\_N\_DM}) \times (Z_S + Z_{OP\_P\_DM} + Z_G)} \tag{S10}$$

$$\begin{aligned} I_R &= \frac{(-Z_S - Z_{OP\_P\_DM} - Z_G) \times V_{NS\_ME\_DM} + (Z_S + Z_{OP\_P\_DM}) \times 1/2\, V_{NS\_ME\_DM}}{(Z_S + Z_{OP\_P\_DM}) \times Z_G + (Z_R + Z_{OP\_N\_DM}) \times (Z_S + Z_{OP\_P\_DM} + Z_G)} \\ &= -\frac{Z_G \times V_{NS\_ME\_DM} + (Z_S + Z_{OP\_P\_DM}) \times 1/2\, V_{NS\_ME\_DM}}{(Z_S + Z_{OP\_P\_DM}) \times Z_G + (Z_R + Z_{OP\_N\_DM}) \times (Z_S + Z_{OP\_P\_DM} + Z_G)} \end{aligned} \tag{S11}$$

Under full symmetry conditions,

$$Z_{OP\_P\_DM} = Z_{OP\_N\_DM} \tag{S12}$$

$$Z_S = Z_R \tag{S13}$$

Thus,

$$I_S = -I_R \tag{S14}$$

Therefore,

$$I_G = 0 \tag{S15}$$

The differential neural signal ($V_{NS\_OP\_DIFF}$) introduced by the operational amplifier (OPA) can be obtained based on the differential-mode signal IC ground as displayed in Figure S4

$$V_{NS\_OP\_DIFF} = V_P - V_N = I_S \times Z_{OP\_P\_DM} - I_R \times Z_{OP\_N\_DM} \tag{S16}$$

Thus,

$$\begin{aligned} V_{NS\_OP\_DIFF} &= \frac{Z_G \times V_{NS\_ME\_DM} + (Z_R + Z_{OP\_N\_DM}) \times 1/2\, V_{NS\_ME\_DM}}{(Z_S + Z_{OP\_P\_DM}) \times Z_G + (Z_R + Z_{OP\_N\_DM}) \times (Z_S + Z_{OP\_P\_DM} + Z_G)} \times Z_{OP\_P\_DM} \\ &+ \frac{Z_G \times V_{NS\_ME\_DM} + (Z_S + Z_{OP\_P\_DM}) \times 1/2\, V_{NS\_ME\_DM}}{(Z_S + Z_{OP\_P\_DM}) \times Z_G + (Z_R + Z_{OP\_N\_DM}) \times (Z_S + Z_{OP\_P\_DM} + Z_G)} \times Z_{OP\_N\_DM} \end{aligned} \tag{S17}$$

Under full symmetry conditions,

$$V_{NS\_OP\_DIFF} = V_{NS\_ME\_DM} \times \frac{Z_{OP\_P\_DM} + Z_{OP\_N\_DM}}{Z_{OP\_P\_DM} + Z_{OP\_N\_DM} + Z_S + Z_R} \tag{S18}$$

2. Impedance quantization

Neural signals are produced by neurons, travel through the electrolyte-electrode interface and interconnect wires, and ultimately reach the amplifier input. Figure S5 presents the impedance range varies for each part of the signal electrode's impedance path based on the Randles model.

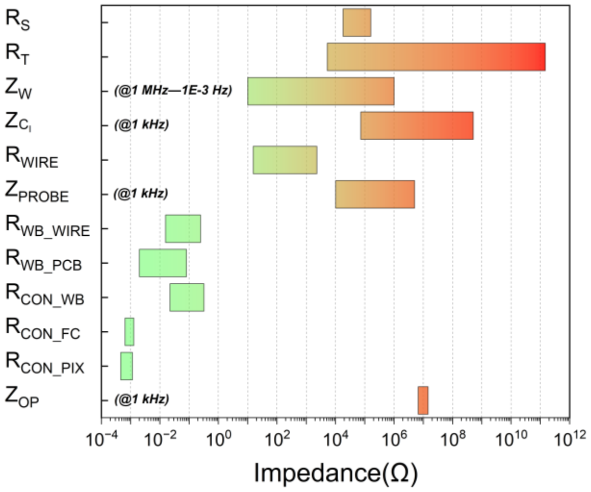

Figure S5. The impedance range varies for each part of the signal electrode's impedance path.

2.1. A. R_S

R_S models the effects of current spreading from a localized electrode to a distant electrode in biological environments. It is regulated by the geometric area and is independent of the effective surface area. For a circular recording electrode with a radius r. The R_S can be calculated as follows[47]:

$$R_{S\_cir} = \frac{\rho_{CSF}}{4r} = \frac{\rho_{CSF}\sqrt{\pi}}{4\sqrt{A_G}} \tag{S19}$$

where A_G is the geometric area of the circular recording electrode. ρ_{CSF} represents the resistivity of the cerebrospinal fluid solution (CSF), measuring 72 Ω·cm when simulated in vitro with phosphate-buffered saline (PBS) solution[48].

For a square recording electrode of length l and width w. The R_S can be expressed as follows[49]:

$$R_{S\_rec} = \frac{\rho_{CSF} \ln\left(4\frac{l}{w}\right)}{\pi l} \tag{S20}$$

To record the extracellular potential of a single neuron, the size of the recording electrode site should be comparable to or even smaller than the single neuron size (1–20 μm). Due to the influence of electrode noise, the minimum recording point area reported so far is 3×1.5 μm2[50]. Therefore, the resistance range of R_S is approximately from 1.803 Ω (circular electrode, r=10 μm) to 1.585 Ω (rectangular electrode, l=3 μm, and w=1.5 μm).

2.2. B. R_T

R_T describes the charge transfer behavior due to the chemical reaction between the electrode and electrolyte, the R_T can be expressed as follows:

$$R_\mathrm{T} = \frac{V_T}{J_0} \cdot \frac{1}{A_S} \tag{S21}$$

where V_T is the thermal voltage, J_0 is the exchange current density between the CSF and electrode, and A_S represents the effective surface area of the electrode. Some published J_0 values include Au with hydrogen reaction (3.98×10^{-6} A/cm2), Ir with hydrogen reaction (2.00×10^{-4} A/cm2), Pt with hydrogen reaction (7.94×10^{-4} A/cm2)[51], and so on. Zhou et al. reported that the current density of bi-continuous conducting polymer hydrogel (BC-CPH) is over 20 times higher than that of a Pt electrode[52]. If a smooth metal is used as the electrode surface, there is little difference between the geometric and effective surface areas. However, the latter can be improved by at least two orders of magnitude using electrode surface modification techniques[53]. Consequently, this work conservatively assumes that the effective surface area of a rough electrode surface is 100 times greater than the geometric area with a smooth electrode surface. The impedance range of R_T is approximately from 5.213 Ω (BC-CPH, circular electrode with a rough surface, r=10 μm) to 1.4511 Ω (Au with hydrogen reaction, rectangular electrode with a smooth surface, l=3 μm, and w=1.5 μm).

2.3. C. Z_W

Warburg proposed that the impedance (Z_W) is based on diffusion. This impedance can be further refined into parallel connections between R_W and C_W[54].

$$Z_W = \left(\frac{1}{R_W} + j2\pi C_W\right)^{-1} \tag{S22}$$

where Z_W can be regulated by adjusting the frequency and effective surface area.

$$|Z_W| = \frac{k}{A_s\sqrt{f}} \tag{S23}$$

where k is a constant dependent on the electrochemistry and mobility of the participating ions, and f is the frequency. For the Pt electrode with a diameter of 20 μm, the impedance of Z_W is approximately from ~101 Ω (at 106 Hz) to ~106 Ω (at 10–3 Hz). This impedance at 1 kHz is approximately 102 Ω[55].

2.4. D. Z_{C_I}

C_I describes the rapid charge accumulation at the electrolyte-electrode interface. This total interface capacitance can be further refined into a series combination of the Helmholtz capacitance (C_H) and Gouy-Chapman capacitance (C_G)[51], as follows:

$$\frac{1}{C_I} = \frac{1}{C_H} + \frac{1}{C_G} = \frac{d_{OHP}}{\varepsilon_0 \varepsilon_r A_S} + \frac{L_D}{\varepsilon_0 \varepsilon_r A cosh\,(\frac{zV_o}{2V_T})} \tag{S24}$$

where $\varepsilon_0/\varepsilon_r$ represents the absolute/relative dielectric constant, d_{OHP} represents the distance between the Outer Helmholtz Plane (OHP) and electrode, L_D is the Debye length, z is the valence of the ions, and V_o represents the potential on the electrode. According to the worst-case estimation of Kovacs, the unit area capacitances C_H/A_S and C_G/A_S are both about 0.14 pF/μm2[49], so the total unit capacitance is about 0.07 pF/μm2. Based on this assumption, the impedance range of Z_{C_I} is approximately from 7.244 Ω (circular electrode with a rough surface, r=10 μm) to 5.068 Ω (rectangular electrode with a smooth surface, l=3 μm, and w=1.5 μm).

2.5. E. R_{WIRE}

The oversight of the impedance (R_{WIRE}) of the metal wires integrated along the probe shafts may weaken efforts to reduce the impedance of the electrolyte-electrode interface. The R_{WIRE} can be expressed as follows:

$$R_{WIRE} = \rho_{WIRE} \frac{l_{WIRE}}{s_{WIRE}} \tag{S25}$$

where ρ_{WIRE} is the electrical resistivity of the metal wire. Generally, gold ($\rho_{Au} = 2.05\ \mu\Omega \cdot cm@23$ ℃) is used as the biocompatibility metal for wiring. l_{WIRE} represents the metal-wire length, which depends on the depth of the implanted brain area. Its interval is set as 1–15 mm, where shallow probes are approximately 1.0–1.5 mm long and depth probes are approximately 2–15 mm long[56]. This interval is obviously the best case because the l_{WIRE} on the headstage is ignored. s_{WIRE} represents the cross section of the wire. There is a trade-off between the cross-sectional area of tissue damage caused by probe implantation and the impedance performance. In s_{WIRE} analysis, the thickness interval is set to 600–3000 Å[57], ignoring the influence of the adhesion layer. The linewidth interval is set to 1–5 μm[58, 59]. Accordingly, the impedance range of R_{WIRE} is approximately from 13.7 Ω ($l = 1\,mm, w = 5\,\mu m, t = 3000$ Å) to 5.1 Ω ($l = 15\,mm, w = 1\,\mu m, t = 600$ Å).

2.6. F. Z_{PROBE}

The current commercial neural recording probe impedance (Z_{PROBE}) combining the R_{WIRE} and Z_{I_B-P}, ranges from 5 kΩ at 1 kHz to 2.5 MΩ at 1 kHz[60].

2.7. G. R_{CON}

As the number of recording channels refreshes to 65536[61], achieving stable interconnection between probes and ASICs while meeting the requirements of a small size cannot be ignored. Similar to (S9), the R_{CON} can be expressed as follows:

$$R_{CON} = \rho_{CON} \frac{l_{CON}}{s_{CON}} \tag{S26}$$

Stable wire bonding (WB) assembly technology with a printed circuit board (PCB) or a flexible printed circuit (FPC) board interconnection scheme is widely used[59, 62]. Regarding the WB impedance, we consider different WB wire materials (Au/Au alloy, Ag alloy, Cu/Cu alloy, Al/Al alloy), WB wire diameters (18–25 μm), and bonding lengths (400 μm–2 mm)[63]. The impedance range of R_{WB_WIRE} is from ~10^{-2} to ~10^{-1} Ω. Regarding PCB or FPC resistance, we estimated copper wiring with a thickness of 1–4.5 OZ, a width of 4–

12 mil, and a length of 5–15 mm. The impedance of R_{WB_PCB} ranges from ~10^{-3} to ~10^{-2} Ω. In summary, the impedance range of R_{CON_WB} ranges from ~10^{-2} to ~10^{-1} Ω.

Compared with WB assembly technology, flip-chip assembly technology based on ASIC die and probe greatly reduces packaging size[64]. Considering the impedance interval of this bonding technology, we consider Sn96.5/Ag3.0/Cu0.5 or Sn42/Bi58 as the bonding solder ball[65], a rectangular ASIC I/O pad with a length and width of 60/50 μm as the bonding pad and 20–30 μm as the bonding height. Accordingly, the impedance of R_{CON_FC} ranged from ~10^{-3} to ~10^{-2} Ω.

The neuropixel probe[66], prepared by CMOS and CMOS-compatible post-processing technology, significantly reduces the impedance sum of traditional R_{WIRE} and R_{CON}. Considering TiN ($\rho_{TiN} = 25\ \mu\Omega \cdot cm$@23 ℃) as the via material, with 5 μm length, 5 μm width, and 800 nm height, and R_{CON} is ~10^{-3} Ω.

2.8. H. Z_{OP}

For capacitively coupled nerve amplifiers, the input capacitance value cannot be very small because of equivalent input impedance-equivalent input noise and equivalent input impedance-closed loop gain. The minimum input capacitance is 9.7 pF[67], and the impedance (at 1 kHz) range is ~13.7–14.9 MΩ, considering the parasitic capacitance (10%–20%). Meanwhile, the maximum input capacitance is 20 pF[68], and its impedance (at 1 kHz) range is ~6.6–7.2 MΩ. Therefore, the impedance range (at 1 kHz) of Z_{OP} is approximately from 6.6 to 14.5 MΩ.

3. Design of the electrodes

Estimating the impedance of brain tissue is a complex process due to various factors, including tissue type, structure, pathological state, measuring frequency, etc. As a result, estimating the impedance of the brain tissue has individual differences and dynamic variability. Currently, there is no universal formula or method to estimate the impedance of the brain tissue. To guide electrode site design using the impedance relationship between electrodes, it is assumed that the closer the distance between electrodes, the lower the impedance of brain tissue based on the traditional Ohm's law.

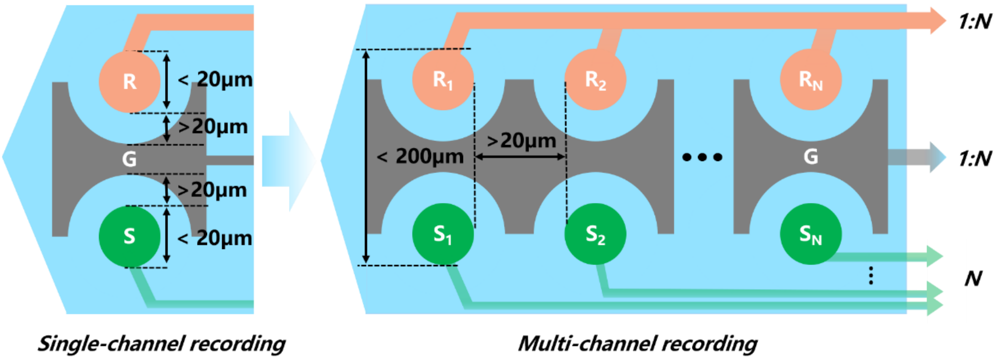

Figure S6. Optimal design of the electrodes in a multiple-channel neural recording system. S, signal electrode; R, reference electrode; G, ground electrode.

To avoid weakening spike caused by the close distance between electrodes, the distance between any two types of electrodes in recording, reference, and ground electrode should be 20–200 μm (Minimum LFP local). The smaller the distance, the smaller the LFP magnitude, but the smaller the EMI magnitude. EMI includes CMI and DMI.

The CMI introduced by OPA is related to both the configuration of the electrodes at the electrolyte-electrode interface and path impedance matching. To eliminate the influence of the interface, the path impedance Z_G should be as small as possible, so the geometric area of the ground electrode (A_{G_G}) should be as large as possible. The inter-electrode impedance Z_{SG} and Z_{RG} should be as small as possible, so the ground electrode site is as close to the signal and/or reference electrode site as possible. Regarding the rigorous

path impedance, it is important to ensure $Z_R = \frac{Z_S}{N}$. When expanding from single-channel recording to multi-channel (N) recording, the geometric area relationship between the signal (A_{G_S}) and reference (A_{G_R}) electrode is $A_{G\_R} = N \times A_{G\_S}$.

The DMI introduced by OPA is related to both the design of the electrodes at the electrolyte-electrode interface and path impedance mismatch. To eliminate the influence of the interface, the signal electrode should be as close as possible to the reference electrode to meet $I_{D\_SG} \approx I_{D\_SG}$. The ground electrode needs to be located at the midpoint of the signal and reference electrodes to meet $Z_{SG} \approx Z_{RG}$. The significance of minimizing CMI from the electrolyte-electrode interface is not only beneficial for the subsequent implementation of common-mode suppression on ASIC but also for reducing the conversion of CMI into DMI due to impedance mismatch (i.e., $\Delta\left(\frac{Z_{OP\_P\_CM}}{Z_{OP\_P\_CM}+Z_S} - \frac{Z_{OP\_N\_CM}}{Z_{OP\_N\_CM}+Z_R}\right)$). Based on the summary of electrode design in the equivalent circuit model, please refer to Figure S6.

References

1. Newman, J. Resistance for Flow of Current to a Disk. *J. Electrochem. Soc.* **1966**, *113*, 501.
2. Chung, T.; Wang, J.Q.; Wang, J.; Cao, B.; Li, Y.; Pang, S.W. Electrode Modifications to Lower Electrode Impedance and Improve Neural Signal Recording Sensitivity. *J. Neural Eng.* **2015**, *12*, 056018.
3. Stenger, D.A.; McKenna, T.M. Enabling Technologies for Cultured Neural Networks; Academic Press, 1994.
4. Guan, S.; Wang, J.; Gu, X.; Zhao, Y.; Hou, R.; Fan, H.; Zou, L.; Gao, L.; Du, M.; Li, C.; et al. Elastocapillary Self-Assembled Neurotassels for Stable Neural Activity Recordings. *Sci. Adv.* **2019**, *5*, 11.
5. Borkholder, D. *Cell Based Biosensors Using Microelectrodes*; Stanford University: Stanford, CA, USA, 1998.
6. Zhou, T.; Yuk, H.; Hu, F.; Wu, J.; Tian, F.; Roh, H.; Shen, Z.; Gu, G.; Xu, J.; Lu, B.; et al. 3d Printable High-Performance Conducting Polymer Hydrogel for All-Hydrogel Bioelectronic Interfaces. *Nat. Mater.* **2023**, *22*, 895–902.
7. Robinson, D.A. The Electrical Properties of Metal Microelectrodes. *Proc. IEEE* **1968**, *56*, 1065–1071.
8. Warburg, E. Ueber Das Verhalten Sogenannter Unpolarisirbarer Elektroden Gegen Wechselstrom. *Ann. Phys.* **1899**, *303*, 493–499.
9. Fontes, M.B.A. Electrodes for Bio-Application: Recording and Stimulation. *J. Phys. Conf. Ser.* **2013**, *421*, 012019.
10. Obidin, N.; Tasnim, F.; Dagdeviren, C. The Future of Neuroimplantable Devices: A Materials Science and Regulatory Perspective. *Adv. Mater.* **2020**, *32*, 26.
11. Park, S.Y.; Na, K.; Vöröslakos, M.; Song, H.; Slager, N.; Oh, S.; Seymour, J.; Buzsáki, G.; Yoon, E. A Miniaturized 256-Channel Neural Recording Interface with Area-Efficient Hybrid Integration of Flexible Probes and Cmos Integrated Circuits. *IEEE Trans. Biomed. Eng.* **2022**, *69*, 334–346.
12. Yang, J.C.; Du, M.D.; Wang, L.; Li, S.X.; Wang, G.R.; Yang, X.L.; Zhang, L.J.; Fang, Y.; Zheng, W.F.; Yang, G.; Jiang, X.Y. Bacterial Cellulose as a Supersoft Neural Interfacing Substrate. *Acs Appl. Mater. Interfaces* **2018**, *10*, 33049–33059.
13. Norlin, P.; Kindlundh, M.; Mouroux, A.; Yoshida, K.; Hofmann, U.G. A 32-Site Neural Recording Probe Fabricated by Drie of Soi Substrates. *J. Micromechanics Microengineering* **2002**, *12*, 414–419.
14. Science, Microprobes for Life. Array Comparision Chart. Available online: https://www.microprobes.com/support-and-download/array-comparison-chart (accessed on 10 June 2024).
15. NEURACOM. Mpk3232a Series. Available online: http://www.neuracom.com.cn/portal/article/index.html?id=108&cid=8 (accessed on 10 June 2024).
16. Blanche, T.J.; Spacek, M.A.; Hetke, J.F.; Swindale, N.V. Polytrodes: High-Density Silicon Electrode Arrays for Large-Scale Multiunit Recording. *J. Neurophysiol.***2005**, *93*, 2987–3000.
17. TANAKA. Bonding Wires. Available online: https://www.tanaka.com.cn/products/detail/bonding-wires/?nav=use (accessed on 10 June 2024).
18. Wang, Q.; Wang, G.; You, C.; Zhang, X.; Liu, D.; Zeng, H.; Xue, N.; Yao, L.; Li, T. An Expandable 36-Channel Neural Recording Asic with Modular Digital Pixel Design Technique. *Electron. Lett.* **2023**, *59*, e12765.
19. ICM Industrial Development Co., Ltd. Bga Solder Ball: Sn42bi58. Available online: https://www.icmrop.com/uploads/attachment/pdf/20220523/TDS-ICM-Sn42Bi58.pdf (accessed on 10 June 2024).
20. Lopez, C.M.; Andrei, A.; Mitra, S.; Welkenhuysen, M.; Eberle, W.; Bartic, C.; Puers, R.; Yazicioglu, R.F.; Gielen, G.G.E. An ImplanTable 455-Active-Electrode 52-Channel Cmos Neural Probe. *IEEE J. Solid-State Circuits* **2014**, *49*, 248–261.
21. Chen, C.H.; McCullagh, E.A.; Pun, S.H.; Mak, P.U.; Vai, M.I.; Mak, P.I.; Klug, A. An Integrated Circuit for Simultaneous Extracellular Electrophysiology Recording and Optogenetic Neural Manipulation. *IEEE Trans. Biomed. Eng.* **2017**, *64*, 557–568.
22. Majidzadeh, V.; Schmid, A.; Leblebici, Y. Energy Efficient Low-Noise Neural Recording Amplifier with Enhanced Noise Efficiency Factor. *Ieee Trans. Biomed. Circuits Syst.* **2011**, *5*, 262–271.
